# Large-Volume Intrathecal Administrations: Impact on CSF Pressure and Safety Implications

**DOI:** 10.3389/fnins.2021.604197

**Published:** 2021-04-14

**Authors:** Vasily Belov, Janine Appleton, Stepan Levin, Pilar Giffenig, Beata Durcanova, Mikhail Papisov

**Affiliations:** ^1^Massachusetts General Hospital, Boston, MA, United States; ^2^Harvard Medical School, Boston, MA, United States; ^3^Shriners Hospitals for Children–Boston, Boston, MA, United States

**Keywords:** cerebrospinal fluid, intracranial pressure, intrathecal delivery, macromolecules, central nervous system, craniospinal compliance, safety, subarachnoid

## Abstract

The increasing number of studies demonstrates the high potency of the intrathecal (IT) route for the delivery of biopharmaceuticals to the central nervous system (CNS). Our earlier data exhibited that both the infused volume and the infusion rate can regulate the initial disposition of the administered solute within the cerebrospinal fluid (CSF). This disposition is one of key factors in defining the subsequent transport of the solute to its intended target. On the other hand, fast additions of large volumes of liquid to the CSF inevitably raise the CSF pressure [a.k.a. intracranial pressure (ICP)], which may in turn lead to adverse reactions if the physiologically delimited threshold is exceeded. While long-term biological effects of elevated ICP (hydrocephalus) are known, the safety thresholds pertaining to short-term ICP elevations caused by IT administrations have not yet been characterized. This study aimed to investigate the dynamics of ICP in rats and non-human primates (NHPs) with respect to IT infusion rates and volumes. The safety regimes were estimated and analyzed across species to facilitate the development of translational large-volume IT therapies. The data revealed that the addition of a liquid to the CSF raised the ICP in a rate and volume-dependent manner. At low infusion rates (<0.12 ml/min in rats and <2 ml/min in NHPs), NHPs and rats displayed similar tolerance patterns. Specifically, safe accommodations of such added volumes were mainly facilitated by the accelerated pressure-dependent CSF drainage into the blood, with I stabilizing at different levels below the safety threshold of 28 ± 4 mm Hg in rats and 50 ± 5 mm Hg in NHPs. These ICPs were safely tolerated for extended durations (of at least 2–25 min). High infusion rates (including boluses) caused uncompensated exponential ICP elevations rapidly exceeding the safety thresholds. Their tolerance was species-dependent and was facilitated by the compensatory role of the varied components of craniospinal compliance while not excluding the possibility of other contributing factors. In conclusion, large volumes of liquids can safely be delivered via IT routes provided that ICP is monitored as a safety factor and cross-species physiological differences are accounted for.

## Introduction

The IT drug delivery route is of increasing interest due to the absence of continuous barriers between the cerebrospinal fluid (CSF) and central nervous system (CNS) (Davson et al., 1987), which makes the fraction of CSF-borne compounds delivered to the brain from the CSF through perivascular channels (Wagner et al., 1974; Rennels et al., 1985; Durcanova et al., 2019) pharmacologically significant (Kakkis et al., 2004; Ishigaki et al., 2007; Tsai et al., 2007; Calias et al., 2011; Papisov et al., 2012a, b). This is especially important for macromolecules, including biopharmaceuticals, for which the access to CNS from the systemic circulation is particularly hindered due to large molecular size and the presence of several vascular barriers (blood-brain, blood-arachnoid, and blood-CSF) (Begley, 2004; Pardridge, 2005).

In the clinical setting, the most commonly used routes for accessing the CSF are: (1) the intrathecal lumbar (ITL) route through the lumbar cistern (enveloping the cauda equina) and (2) the more invasive intracerebroventricular (ICV) route through the brain parenchyma into one of the lateral ventricles of the brain. Although the ICV route is clinically feasible, it requires the surgical installation of a transcranial port with an accompanying cannula (Ommaya reservoir). The ITL route is significantly less invasive and can be utilized for chronic use (with surgical installation of subcutaneous ports or electronic pumps) as well as for one-dose injections directly through interspinous ligaments in the L3–L5 region. Both methods are relatively simple, well developed, and particularly suitable for biopharmaceuticals, such as gene vectors, intended for single injections or multiple injections given at a large interval (several months).

The ITL route is commonly used in pain and spasticity management for chronic delivery of respective drugs from subcutaneously implanted electronic pumps. Such pumps slowly deliver very small volumes directly to the target (nerve roots). The action of the drug delivered in such a way is intended to be local, without excessive rostral spread, which is achieved by controlling the hydrophilic/lipophilic properties as well as administration rate and volume of the solute. Despite being well-established in clinic, this approach, unfortunately, does not address the delivery demands for CNS-targeted biopharmaceuticals (Papisov et al., 2012b). Specifically, evoking a pharmacologically relevant effect at CNS targets in the brain or spinal cord, or both requires distribution of high concentrations of the IT-injected compound through the majority of CSF compartments and perivascular spaces at high rates to address the concurrent clearance into the blood. Our data obtained earlier in non-invasive positron emission tomography (PET) studies suggested the absence of directional CSF flows within the spine (Papisov et al., 2012b, 2013). We also found that the ITL administration of a larger volume (up to 30–50% of the total CSF volume) caused immediate translocation of the administered solute to the cerebro-cervical area, resulting in a nearly identical initial disposition in the CSF as compared to ICV administration (Calias et al., 2011). Rieselbach et al. (1962) demonstrated that both humans and non-human primates (NHPs) could tolerate large-volume ITL boluses of up to 33 and 42% of the total CSF volume, respectively. Therefore, the ITL route is currently of significant interest for testing the efficacy of novel biopharmaceuticals in pre-clinical models as well as for clinical use. However, the physiological mechanisms responsible for the accommodation of such additional large volumes by the subarachnoid (leptomeningeal) space as well as the safety limits of different IT administration regimes remain insufficiently characterized.

Due to the confinement of the subarachnoid liquid-filled space by rigid (bone) boundaries, the modified Monroe-Kellie doctrine (Mokri, 2001) states that the total volume of its contents must remain constant. The brain, CSF, and blood are the major constituents, the volume fractions of which under normal conditions are 83, 11, and 6%, respectively. Given the constant volume of the brain tissue, it is the hydrodynamic balance of blood and CSF volumes that primarily determines the CSF pressure [often referred to as intracranial pressure (ICP)]. In this respect, experimentally measurable characteristics of ICP can be evaluated as safety factors characterizing the impact of IT administrations.

Intracranial pressure is not constant and on a long-term scale (hours), depends on the balance of CSF production and drainage. On a shorter-term scale (seconds), ICP fluctuates due to the rhythmic changes of the cerebral blood volume (and respiratory fluctuations). Such a dynamic nature of the ICP signal necessitates analysis of not only the mean value, but also of the ICP waveform (Czosnyka and Pickard, 2004; Kawoos et al., 2015). The latter is characterized by periodic pulses each consisting of three notches P1, P2, and P3, progressively decreasing in amplitude and reflecting propagation of the arterial pulse pressure wave. P1 harmonic is a fundamental component (percussion wave) that represents transmission of the arterial pulse through the choroid plexus and other highly vascularized tissues, such as cerebellum and major arteries interfacing the CSF, to the CSF. The amplitude of this component and the average ICP are the key parameters used for characterizing the ICP autoregulatory capacity i.e., the ability to maintain a normal ICP. This capacity can be assessed by studying the ICP-CSF volume and the ICP amplitude (P1)-mean ICP relationships. There is significant evidence indicating the successful use of these relationships for deriving indices effective for the diagnosis and assessment of treatments for neurological disorders affecting the liquid homeostasis in the CNS such as subarachnoid and intracerebral hemorrhage, ischemic stroke, hydrocephalus, meningitis/encephalitis, and traumatic brain injury among others (Avezaat et al., 1979; Czosnyka and Pickard, 2004; Hawthorne and Piper, 2014; Kawoos et al., 2015).

Cerebrospinal fluid drainage into blood plays a vital and primary role, as evidenced by our imaging studies (Calias et al., 2011; Papisov et al., 2012a, b), in maintaining a healthy hydrodynamic balance in the CNS and in adaptation to elevated ICPs due to the pressure-valve function of the drainage sites. The vast majority of these sites are located at the CSF-blood interface in the arachnoid protrusions into the venous sinuses (arachnoid granulations/villi) within the cranial (superior sagittal and transverse venous sinuses) and spinal (venous sinuses near dorsal root ganglia) compartments (Kapoor et al., 2008; Pollay, 2010). Significant progress has been made in establishing both the theoretical and experimental relationships between ICP and CSF outflow dynamics through CSF infusion tests for using as a clinical tool for the assessment of normal pressure hydrocephalus and shunt functionality (Eklund et al., 2007; Sundström et al., 2010). However, the utility of the developed methods for IT drug delivery has not yet been evaluated.

In humans, 7–15 mm Hg is considered a normal range of ICP as measured via lumbar access in supine adults (Albeck et al., 1991). While ICP in humans is commonly reported for the supine position, the measured value is known to be a function of a body posture (Czosnyka and Pickard, 2004). For example, in the vertical position, ICP baseline level is negative with a mean of around −10 mm Hg, but not exceeding −15 mm Hg (Chapman et al., 1990). Although tolerable, persistently elevated ICP levels of 15–22 mm Hg are considered hypertensive and are indicative of an underlying pathology requiring treatment (Kawoos et al., 2015). Aggressive therapy is typically initiated when ICPs surpass 22 mm Hg (Czosnyka and Pickard, 2004; Carney et al., 2017). These pressures are characteristic of impaired autoregulatory capacity and if present for sustained periods of time [>37 min in adults and >8 min in children (Guiza et al., 2015)], may be predictive of poor patient outcomes in regards to survival rates and a long-term functionality (Czosnyka and Pickard, 2004). Moreover, the modeled relationship between elongated periods of high ICPs and poor outcomes was found to be nearly exponential in both the adult and pediatric populations (Guiza et al., 2015). ICP levels exceeding 40 mm Hg are characteristic of acute brain injuries and cannot be tolerated for extended durations (>30 min) (Castellani et al., 2009; Kawoos et al., 2015). It should be noted that reported ICP thresholds are even lower for special populations such as the pediatric, elderly, and female populations (Sorrentino et al., 2012; Guiza et al., 2015). One of the major factors associated with adverse events of prolonged elevated ICP is the cerebral perfusion flow, critical decrease of which can cause secondary ischemic injury (Kawoos et al., 2015). Cerebral perfusion flow is regulated by the cerebral perfusion pressure (CPP) defined by the difference between mean arterial pressure and ICP (Kirkman and Smith, 2014; Czosnyka et al., 2017). The current guidelines recommend maintaining CPP between 60 and 70 mm Hg (Carney et al., 2017). This approach aims to prevent secondary injuries due to hypoperfusion (e.g., cerebral ischemia) or hyperperfusion (e.g., edema) (Kawoos et al., 2015). Increased ICP levels also adversely impact the brainstem function as excessive pressures can cause bradycardia and hypertension (Cushing reflex), and if left untreated, precipitate respiratory depression and death (Smith, 2008).

Based on the clinical observations, the extent of adversity to large-volume IT injections/infusions should depend not only on the magnitude of the CSF pressure elevation but also the duration of such increases. This fact implies that both the volume and the delivery rate of liquids added to the CSF contribute to defining the safety thresholds provided that the relationship between these factors and the CSF pressure is established. These thresholds, least the physiological mechanisms underlying them, have not been studied systematically with respect to IT administrations, in neither humans nor laboratory animals.

Considering prior advances in ICP research obtained primarily from trauma and hydrocephalus patients, this study aims to establish a method of ICP monitoring for characterizing disbalances in the CSF pressure caused by various modes of IT administrations. The safety thresholds are estimated and analyzed across species to provide recommendations for translational studies aiming at developing clinically relevant approaches for monitoring the safety of IT therapy delivery. To this end, we carried out experiments in rats and NHPs and: (1) determined the relationship between ICP, injection rates and volumes, (2) characterized the safety thresholds for ICP elevations, and (3) investigated the patterns of ICP relaxation to the baseline levels. The obtained data are discussed within the context of current knowledge on the involved physiological mechanisms and translational implications.

## Materials and Methods

### Animals

All animal studies were reviewed and approved by the Institutional Animal Care and Use Committee of Massachusetts General Hospital. Male Sprague-Dawley CD rats (*n* = 8, 325 ± 76 g) were obtained from Charles River Laboratories (Shrewsbury, MA, United States). One cynomolgus (*Macaca fascicularis*, 7.5 kg) and one rhesus monkey (*Macaca mulatta*, 8.2 kg) were obtained from Northern Biomedical and were equipped with a subcutaneous injection port (P.A.S. Port *Elite*, Smiths Medical ASD, Inc., internal volume: 298 μl). The port was connected to a polyurethane kink-resistant catheter (Smiths Medical ASD, Inc.) entering the subarachnoid space at the L4/L5 spinal segment and advanced to the L1/T12 area. The catheter specifications were as follows: proximal internal diameter: 1.07 mm, proximal outside diameter: 1.93 mm, distal internal diameter: 0.53 mm, distal outside diameter: 0.86 mm, length: 35 cm, volume: 104 μl. The port in each monkey was surgically implanted more than 3 months prior to the study and maintained as recommended per the manufacturer. Catheter patency and absence of leaks were confirmed by PET imaging with macromolecular tracers labeled with ^89^Zr, as in our previous studies (Calias et al., 2011; Papisov et al., 2012a, b). By the time of these studies, the ports had one-way (inwards) patency, likely due to the development of tissue flaps around the catheter openings. All animals were kept on a 12 h/12 h light/dark cycle, switching on at 7 AM and off at 7 PM. Food and water were provided *ad libitum* to rats. Monkeys had three meals a day while the water was provided *ad libitum*.

### ICP Registration Methodology

Intracranial pressure in rats was measured directly in the cisterna magna pool of the CSF using a 1F piezoresistive diffused semiconductor pressure sensor mounted on the tip of a 20 cm long 0.8 F polyimide catheter (Millar, Inc.). The pressure catheter was inserted through a temporary angiocatheter installed through the atlanto-occipital membrane. In monkeys, port configuration did not allow sensor insertion into the leptomeningeal space. The pressure (ICP_*L*_) was measured externally, using the same sensor inserted into a saline-filled catheter line connected to the subcutaneous IT port. Electrical signals from the sensor were processed using a FE221 Bridge Amplifier (ADInstruments) coupled with a PowerLab 4/35 data acquisition platform (ADInstruments). Control of both modules, signal acquisition, and subsequent ICP waveform analysis were performed using a LabChart 8 software (ADInstruments) installed on a MacBook Pro laptop (Apple, Inc.). ICP sampling rate was set to 1000 measurements per second. Prior to all ICP measurements, the pressure transducer was soaked in sterile water or saline at room temperature for at least 30 min. After that, atmospheric pressure was set to 0 mm Hg and the stability of the sensor signal was ensured by recording the baseline for at least 10 min.

### Rats

#### Cisterna Magna Catheterization

Percutaneous non-surgical catheterization of the cisterna magna was carried out following the technique developed by Jeffers and Griffith (Farris and Griffith, 1949). Animals were anesthetized by inhalation of isoflurane/air mixture (3% for induction, 2% for maintenance) given at 300 ml/min flow rate using a SomnoSuite^®^ small animal anesthesia platform (Kent Scientific). The animal was then mounted on a stand inclined to 50° angle with the head flexed down, thereby positioning the occipital bone in the horizontal plane. The back of the neck and the base of the skull were shaved and disinfected with 70% ethyl alcohol. The location of the cisterna magna was identified by palpation of a 3-mm^2^ rhomboid depressed area (atlanto-occipital joint) in the middle of the line connecting the ear bases. A 24G 0.75” IV catheter (Angiocath^TM^, Becton Dickinson) was then slowly inserted to a depth of 7 mm. The inner needle was immediately removed after the appearance of the clear CSF in the outer catheter. The catheter’s Luer adapter was then promptly capped, and the catheter was affixed to the skin with a cyanoacrylate gel. Localization of the tip of the (radiopaque) catheter in the cisterna magna was verified at the end of the study by taking a CT scan of a rat’s head (Supplementary Figure 1A).

#### ICP Measurements

Intracranial pressure measurements in rats were carried out using the equipment configuration shown in Supplementary Figure 1B. Specifically, after the cisterna magna catheterization, a Tuohy Borst adapter (Cook Medical) equipped with a side arm (initially closed) and pre-filled with saline was connected to the IV catheter via a Luer adapter. A micro-tip pressure transducer was then slowly advanced into cisterna magna through the entire length of the Tuohy Borst adapter and the IV catheter. The adaptor was tightened up, and the baseline ICP referenced to the atmospheric pressure (pre-set to 0 mm Hg) was recorded for 5–15 min. One of the following procedures was subsequently performed.

##### ICP setting

A 60” extension set polyvinyl chloride minibore tubing (Acacia) connected to a bottle of sterile saline and subsequently primed was attached to the Tuohy Borst’s side arm. ICP registration started immediately after opening the side arm. Initially, the saline surface meniscus was leveled with the cisterna magna thereby equilibrating ICP with the ambient atmospheric pressure. ICPs of 1.0 ± 0.4, 5.5 ± 1.1, 8.3 ± 2.5, 12.3 ± 0.7, 17.6 ± 1.2, 21.1 ± 2.7, 24.7 ± 3.8, 30.1 ± 5.1, and 37.0 ± 2.3 mm Hg were then set in each of the four rats by positioning the saline bottle at progressively increasing heights. At each height, ICP was recorded for 10–15 min.

##### CSF drainage assessment

A sterile saline bag was suspended on a bench-top pole and attached to a dripper. The dripper was a manually made device for the flow rate measurement. It consisted of a conical plastic tip hermetically inserted inside the air-filled plastic cylinder. The outside end of the tip was used for the attachment to the saline bag. The opposite end of the cylinder was connected to the saline line leading to the side arm of the Tuohy Borst adapter. When the saline flow was activated by placing a saline bag at different heights, the dripper produced drops of calibrated size (12.8 ± 0.2 ml), the number of which was visually counted using a timer thereby enabling a flow rate calculation. ICP fluctuations caused by falling drops (Figure 2A) provided an additional method for the dripping rate measurement. Changes to the saline column’s heights caused respective changes in the baseline ICP levels ranging from 0 to 50 mm Hg, which were measured using the ICP registration methodology. For each ICP level, continuous recording was performed for 10–15 min. Experiments were repeated in 4 rats weighing 443 ± 104 g.

##### IT infusions with a pump

A Silastic^TM^ silicone tubing (Dow Corning, 0.76 mm inner diameter, 1.65 mm outer diameter), primed and connected to a 1 ml syringe (Becton Dickinson) filled with saline and mounted on a syringe pump (Model 22, Harvard Apparatus), was attached to the Tuohy Borst’s side arm. ICP recording was activated immediately with the opening of the side arm and the start of the infusion of 200–240 μl of saline at one of the following rates: 10, 20, 40, 80, and 120 μl/min. Each rate was tested in three different rats. Each of 4 rats was used for 3–4 serial tests allowing sufficient time between infusions as confirmed by the ICP relaxation to the baseline level. The quality of infusions was controlled by visual examination of the skin puncture site and all connections for the signs of leakages which were absent in all instances.

##### Manual bolus IT injections

An infusion plug (Argyle^TM^, Covidien) equipped with a rubber septum was attached to the Tuohy Borst’s side arm. A 1-ml tuberculin syringe (Becton Dickinson) filled with saline was inserted in the plug and the injection of a tested volume was performed at 2.7 ± 0.9 ml/min. This is the rate that was typically used in our previous studies utilizing manual cisterna magna injections. The syringe was withdrawn at the end of the injection, and the ICP was allowed to return to the baseline level. The syringe was reloaded, and the injection of the next volume was performed. The following volumes were tested: 0.05, 0.1, 0.15, 0.2, 0.25, 0.3, 0.35, 0.45,0.6, 0.8, 1.0, and 1.5 ml. For the injection of 1.5 ml of saline, a 3 ml syringe equipped with a 23G needle (Becton Dickinson) was used. The quality of injections was controlled by visual examination of any residuals in the injection syringe as well as the skin puncture site and all connections for the signs of leakages which were absent in all instances. A temperature-controlled thermal pad or a heating lamp maintained body temperature over the entire duration of the procedures (ca. 2 h).

### Monkeys

#### Sedation and Preparation

Animals were fasted for 12 h before the experiment. Atropine sulfate (0.03 mg/kg) was administered intramuscular (IM) 1 h before the study to suppress saliva production. 10 min thereafter, the animal was sedated with IM injection of Ketamine (15 mg/kg)/Xylazine (1 mg/kg) at the animal facility and transported to the laboratory. The animal was intubated with a 3 mm (∅) endotracheal tube (Mallinckrodt^TM^, Covidien), and the tube was connected to the anesthesia line providing continuous 1.5–2% isoflurane/O_2_ flow at 2 l/min. Heart and respiration rates, blood oxygen saturation (SpO_2_), end-tidal CO_2_ content, and rectal temperature were monitored continuously and documented every 15 min. Isoflurane content and flow rate were adjusted upon need to maintain physiological vitals. The anesthetized animal was placed on a soft pad in a prone position. Supplemental heat was provided via a blanket filled with circulating warm water.

#### IT Injections and ICP Measurements

Intracranial pressure measurements in monkeys were carried out using the equipment configuration shown in Supplementary Figure 2. Prior to the experiments, the port patency and implanted catheters/connectors integrity were ensured during regular, dedicated assessments by trained veterinarians as well as using the most recent PET/CT images from the other experiments involving NHPs being tested. The skin above the IT port was shaved and wiped with 70% isopropanol and treated with Betadine. A saline-primed Huber needle (Access Technologies), attached to the t-connector (Microbore extension set–5 inch, Hospira) with a clamped catheter, was inserted into the port. The catheter was then attached to the saline-primed side-armed Tuohy Borst adapter (Cook Medical). 1F micro-tip pressure catheter (Millar, Inc.) was inserted inside the adapter, down to the Y-junction, and leveled with the surface of the animal’s spine. The Tuohy Borst’s side arm was then opened to equilibrate the line pressure with the ambient atmospheric pressure. Once a stable pressure line was recorded for at least 10 min, the Tuohy Borst’s side arm was closed and the connection with the t-connector was opened enabling indirect in-line ICP (ICP_*L*_) recording. Baseline registration was carried out for at least 10–15 min. Continuous ICP monitoring was then performed during the IT administration of one 5 ml saline bolus in the rhesus monkey and five 1 ml saline boluses, 2.5 min apart or two 3 ml saline boluses, 8 min apart, in the cynomolgus monkey. To emulate the conventionally used procedure, trained personnel performed all IT infusions in monkeys manually. Infusion rates were determined by calculating the ratio of the duration of steady advancement of the syringe plunger to the administered volume. The quality of administration was controlled by visual examination of any residuals in the syringe, spillovers or leakages, which were absent in all instances. Upon the study completion, anesthesia was gradually withdrawn and the animal was returned to the housing facility.

### Measurement of Hydrostatic Resistance in the Injection Port (*in vitro*)

The same pressure measurement set-up as in the *in vivo* study with monkeys was utilized. A 23G needle (Becton Dickinson) attached to the 60″ extension set polyvinyl chloride minibore tubing (Acacia) was inserted into the t-connector. The other side of the tubing was attached to the 10 ml syringe (Becton Dickinson) filled with saline and mounted on a syringe pump (Model 22, Harvard Apparatus). All lumens were primed with saline prior to pressure measurements. Infusion at one of the tested rates was initiated and lasted for 107.8 ± 37.3 s until the steady state pressure was established and recorded for 101.0 ± 37.2 s. When the infusion was completed, the pressure was allowed to return to the baseline level. 102.0 ± 42.5 s after the steady baseline recording, infusion at the next rate was performed. The following infusion rates were tested: 0.01, 0.05, 0.1, 0.2, 0.4, 0.6, 0.8, 1.0, 1.5, 2.0, 2.5, 3.0, 3.5, 4.0, 4.5, 5.0, 5.5, 6.0, 6.5, 7.0,7.5, and 8.0 ml/min.

### Post-acquisition ICP Data Processing

Post-acquisition data processing was performed using LabChart 8 software (ADInstruments). Mean ICP was determined by averaging a pressure pulse wave over 4 ± 1 min (1478 ± 387 pulses). ICP amplitude is defined as the amplitude of the fundamental (P1) component of the ICP pulse wave. To determine it, fast Fourier transform (FFT) of the ICP time block used for the mean ICP calculation was performed using the following parameters: FFT size: 1000 or 2000 points, data window type: Welch, window overlap: 93.75%. Spectrally resolved P1 harmonic was identified in the frequency range of 3.9–8.8 Hz. ICP relaxation time characterized elasticity of the anatomical components of intrathecal compliance and was measured as a time required for ICP to decrease by 90% from the peak value. Data fitting was performed by a least squares method using Microsoft Excel 2013 (Microsoft) (linear fitting) or Matlab R2018a (MathWorks) [Curve Fitting Toolbox^TM^ using custom weighted (1/(standard deviation)^2^) sigmoidal (*y* = a/(1 + b^∗^exp(-c^∗^x)) and exponential (two terms) functions].

## Results

### Adaptability of Rats to the Increased ICP

An IT administration normally results in ICP elevations due to the increased volume in the subarachnoid space. Therefore, it was important to investigate how different ICP elevations are tolerated in order to determine safe regimes of IT infusions. To this end, we induced in rats (*n* = 4) progressively increased CSF pressures and monitored the changes in the key ICP parameters: mean ICP, ICP amplitude (P1), and spectral characteristics (frequencies and amplitudes) of the ICP waveform’s major components (harmonics).

**FIGURE 1:**
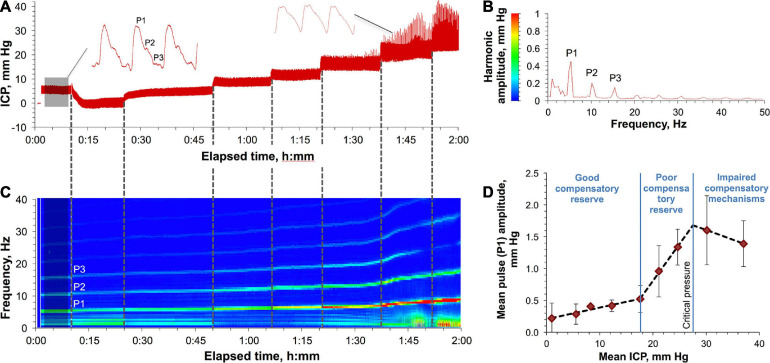
Characteristics of ICP elevation in a rat. **(A)** Various ICPs induced by a saline column attached to the rat’s CSF in the cisterna magna. Shaded area indicates baseline ICP. A representative ICP pulse waveform at low and high pressures are shown in the left and right sides, respectively. P1, P2, and P3 are the major pulse’s harmonics. **(B)** The amplitude-frequency characteristics of the spectrally resolved P1, P2, and P3 harmonics of the baseline ICP pulses (shaded area in panel **A**). A result of averaging of 2564 FFTs each consisting of 2000 points. **(C)** Spectrum view of the changes in the harmonics’ frequencies and amplitudes as a result of ICP elevation over time **(A)**. **(D)** ICP autoregulatory capacity and compensatory reserve in rats. Three specific zones characterizing different states of ICP compensatory reserve are shown and are based on the correlation between the mean amplitude of the P1 component of the ICP pulse and the mean ICP. Each data point represents a mean value obtained by averaging over four animals. Error bars indicate standard deviations. Least-squares linear regression is depicted for each zone with the following R^2^ statistic (left to right): 0.9438, 0.9978, and 1.0000.

Figures 1A–C show a representative ICP monitoring experiment in a rat undergoing a progressive ICP elevation. Baseline ICP (Figure 1A, shaded area) averaging 8.6 ± 1.7 mm Hg (across all studies) was characterized by periodic pulses, a waveform of which had a classical shape (Figure 1A, left waveform) with spectrally resolved P1, P2, and P3 components (Figure 1B). ICP elevations caused waveform distortion (Figure 1A, right waveform) reflecting the changes in the spectral characteristics (frequency and amplitude) of the harmonics as shown in Figure 1C. Specifically, the harmonics’ frequencies and amplitudes, as well as the distance between harmonics increased with ICP elevation, with the most dramatic change affecting the amplitude (5.2 ± 1.5–fold increase). All the changes became rapidly pronounced after the pressure reached 18 mm Hg, before which only a minor yet consistent increase was observed. Spiking artifacts in the waveforms, which became pronounced at ICP > 20 mm Hg (Figure 1A), were caused by the increased neck motion of the rats due to the increased respiration rate (visual observations).

The adaptability of rats to the increased ICP (autoregulatory capacity) was characterized by the relationship between the time-averaged amplitude of the P1 component and the mean ICP (Figure 1D). This relationship has been shown to be a function of the capacity of the compensatory mechanisms to maintain the physiologically relevant pressures in response to different perturbations in the CSF volume (Czosnyka and Pickard, 2004; Hawthorne and Piper, 2014; Kawoos et al., 2015). The least-squares linear regression analysis was employed to analyze correlation between the two variables. R^2^ statistic was used as a metric of fitting performance to segregate three ICP regions with the best outcomes (highest *R*^2^). The detected regions were characterized by: (1) low synchronization (good compensatory reserve) at low ICPs (1–18 mm Hg, *R*^2^ = 0.9438), (2) high synchronization (poor compensatory reserve) at higher ICPs (18–28 mm Hg, *R*^2^ = 0.9978), and (3) negative correlation (impaired compensatory mechanisms) at ICPs > 28 ± 4 mm Hg (“critical pressure”) (*R*^2^ = 1.0000). All rats that underwent ICPs higher than the critical pressure for prolonged time (>10 min) experienced a fatal outcome.

### Performance of the CSF Drainage System Under Pressure

**FIGURE 2:**
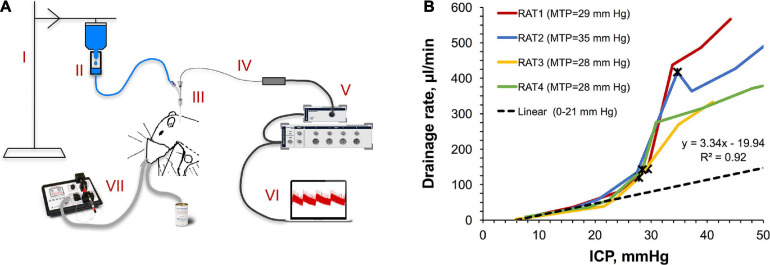
Viability of the CSF drainage system under pressure. **(A)** A sketch of the originally developed experimental setup for measuring the rate of unconstrained CSF drainage at different ICPs. Components of the setup: (I) bench-top pole with a suspended saline bag set at different heights, (II) dripper with calibrated drop size attached to the saline bag and connected to the saline line, (III) Tuohy Borst adapter attached to the catheter inserted in the cisterna magna; saline line connected to the adapter’s side arm, (IV) micro-tip pressure sensor advanced to the cisterna magna through the adapter and catheter, (V) data acquisition platform, (VI) control and data analysis console (note the ICP fluctuations on the computer screen caused by falling drops), (VII) small-animal isoflurane anesthesia platform. **(B)** ICP dependency of the CSF drainage rate. MTP: maximally tolerated pressure. Black asterisks mark MTP for each rat. Data points in the 6–23 mm Hg region were used to build a linear regression (parameters are shown).

Safe accommodation of volumes added to the CSF requires a concurrent engagement of CSF drainage mechanisms. To assess the capacity of those under different ICPs, we developed a novel method to measure the CSF drainage rate schematically depicted in Figure 2A. Briefly, it leverages the measurement of the saline intake in a fluid line connected to the CSF pool, which is challenged by different pressures using a water column effect. Figure 2B illustrates the obtained ICP dependency of the CSF drainage rate for individual rats (*n* = 4). The dependency has a gross exponential shape with a good linearity in the beginning (6–23 mm Hg). A reciprocal to the linearity coefficient denotes the outflow resistance (0.30 mm Hg/(μl/min)) characterizing the drainage performance. Deviations from linearity in the form of lower ICP than expected at ICP > 23 ± 1 mm Hg were consistent with the respective region of weak compensatory mechanisms (18 mm Hg < ICP > 28 mm Hg) shown in Figure 1D. Non-linearity can be explained by altered flow characteristics due to either the change in the capacity of the normal CSF drainage sites or the emergence of additional outflow pathways. Maximally tolerated pressures (MTP) marked with black asterisks averaged 30 ± 3 mm Hg correlating with the region of impaired compensatory mechanisms (ICP > 28 mm Hg) shown in Figure 1D. The following reduction of the drainage rate at ICP > MTP is likely indicative of the discontinuation of some drainage pathways due to death.

### Pump Slow-Rate IT Infusions in Rats

**FIGURE 3:**
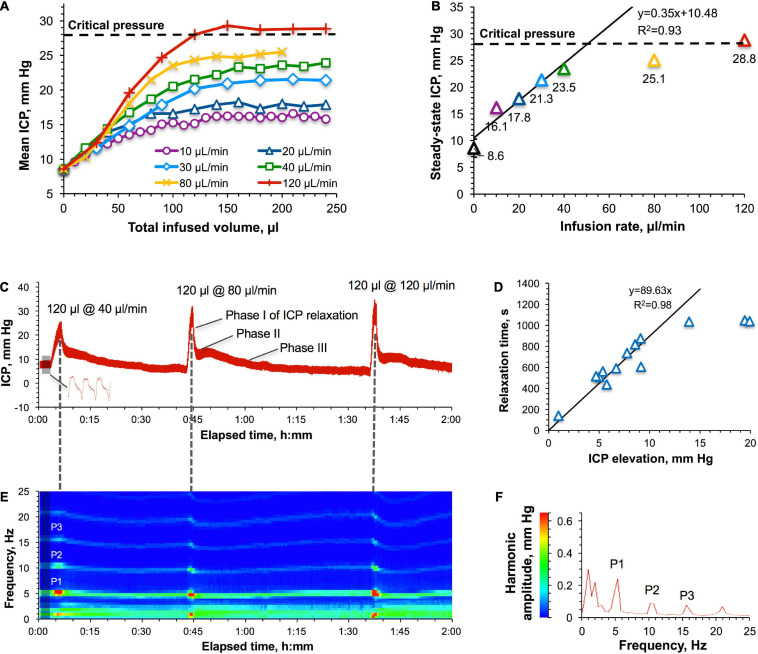
Intracranial pressure (ICP) response to IT infusions. **(A)** ICP–IT infused volume relationship for different infusion rates. “Critical pressure” (28 ± 4 mm Hg) denotes the ICP above which pressure compensatory mechanisms were found to be impaired (Figure 1D). **(B)** Relation of the approximated plateau ICPs with the infusion rates. Parameters of the linear regression for infusion rates in the interval of 10–40 μl/min are shown. Error bars represent standard errors obtained by averaging the data points in Figure 1A at the steady state (>150 μl of the infused volume). **(C)** A representative picture of the ICP response to the infusion of the same volume (120 μl) at three different rates–40, 80, and 120 μl/min. The shaded area is the baseline ICP. A representative ICP waveform is shown in the bottom. **(D)** Relationship between the time of relaxation to the baseline ICP and the magnitude of ICP elevation. Non-averaged data points of individual experiments are shown. Parameters of the linear regression are given for 10–40 μl/min infusion rates. **(E)** A representative picture of the harmonic analysis of the shaded area in panel **(C)**. A result of averaging of 1158 FFTs each consisting of 2000 points. P1, P2, and P3 harmonics were spectrally resolved at all pressures. **(F)** Spectrum view of changes in the harmonics’ frequencies and amplitudes as a result of IT infusions.

Once the critical pressure, above which adaptation to the increased ICP is impaired, was determined, it was used to establish and characterize safe regimes of IT administrations. To this end, we employed a continuous infusion method when the rats (*n* = 4) were infused 200–240 μl of saline (ca. 100% of the total CSF volume in rats) at different rates while the ICP response was continuously monitored. The obtained data demonstrated that the shape of the ICP buildup is irrelevant of the infusion rate in the tested interval of slow rates (10–120 μl/min) (Figure 3A). All rates led to the ICP stabilization. However, despite different plateau levels, ICP steady state occurred at the same infused volume of ca. 150 μl. The relationship of the plateau ICP with the infusion rate (Figure 3B) showed a good linearity up to 23.5 mm Hg (40 μl/min), with the linearity coefficient (0.35 mm Hg/(μl/min)) denoting the outflow resistance. Infusion rates higher than 40 μl/min caused significant deviations from linearity in Figure 3B in the form of lower steady-state ICPs than expected.

We hypothesized that accommodation of liquids added to the CSF at rates <40 μl/min could be achieved by pressure-dependent accelerated drainage of the CSF, whereas compensation of higher-rate infusions would require engagement of an additional mechanism likely based on intrathecal compliance. Assessment of the respective involvement of both mechanisms can be accomplished by analyzing the relaxation of the progressively elevated ICP to the baseline level. For example, 40, 80, and 120 μl/min infusions of 120 μl of saline caused rapid ICP increases above 20 mm Hg followed by the repeated patterns of a multi-phase relaxation process (Figure 3C). Among two or three relaxation phases, the first and the fastest phase (phase I) had an increasing contribution with the pressure increase. This could be the result of the action of the mechanisms of intrathecal compliance due to the significantly limited elasticity capacity of the anatomical correlates (not presently clearly established). Analysis of the dependency of the relaxation time on the ICP elevation magnitude (Figure 3D) obtained for all studies revealed a linear region followed by stabilization at 1044 ± 6 s. The bend point at 12 mm Hg (20.6 mm Hg of the total ICP) corresponding to the infusion rate of 29 μl/min (Figure 3B) could be an indication of the transition from one to two (or more) major mechanisms involved in the accommodation of liquids added to the CSF at raising infusion rates. Indeed, all infusions that caused ICP elevations exceeding 12 mm Hg were followed by multi-phase relaxations.

Intracranial pressure waveform and frequency analyses of the ICP curve in the representative example given in Figure 3C revealed the presence of P1, P2, and P3 harmonics that remained at their characteristic frequencies (5.4, 10.3, and 15.6 Hz, respectively) (Figure 3F) during ICP elevation and relaxation phases (Figure 3E). This observation contrasts with constant-pressure studies (Figure 1C), which is likely to be explained by the shorter infusion time (1–3 min) seen in the latter case, which was presumably insufficient to increase the heart rate in response to the elevated ICP. The harmonics’ amplitudes, in contrast, promptly increased with each ICP elevation (Figure 3E, color transitions from green to red) suggesting the robust impact of these infusions on the compliance-based compensatory reserve, which agrees with our findings shown in Figure 1D (poor compensatory reserve region).

Time-dependency of the response of different physiological components to elevated ICP turned out to play a critical role in the safety of large-volume IT boluses as follows below.

### Manual Bolus IT Injections in Rats

**FIGURE 4:**
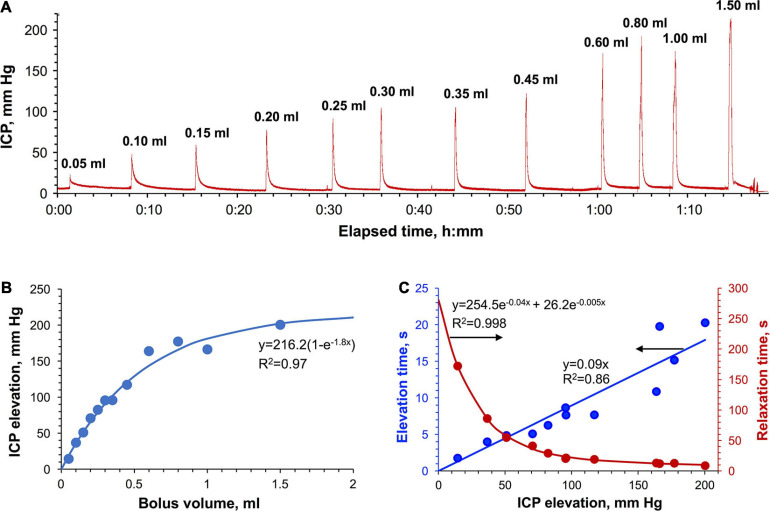
Bolus IT injections in rats. **(A)** The time-dependence curve of ICP elevation. The average administration rate is 2.7 ± 0.9 ml/min. **(B)** Dependence of the ICP elevation on the injected volume. **(C)** The pressure dependence of the total ICP elevation (blue) and ICP relaxation time (red).

Sequential injections of saline volumes ranging from 0.05 to 1.5 ml at the average rate of 2.7 ± 0.9 ml/min in the cisterna magna of a rat resulted in the prompt ICP elevations (Figure 4A). The dependence of the magnitude of those elevations on the injected volume had a logarithmic shape (Figure 4B). ICP stabilized at ca. 200 mm Hg level, which was found to be the maximally tolerable threshold suggesting the capacity limit of the relevant adaptation mechanisms. ICP relaxations to the baseline level (Supplementary Figure 3) were of bi-exponential shape as evidenced by moving frame linearizations [Guggenheim method (Guggenheim, 1926)] having a bi-linear shape (Supplementary Figure 4). Bi-exponentiality reflects the involvement of fast and slow relaxing components characterized by large and small time constants. Relative fractions of both components as a function of the ICP elevation magnitude are shown in Supplementary Figure 9A revealing the diminished contribution of the slow relaxation processes as the ICP increased. It is of note that the relationship of the total relaxation time (ranging from 9 to 172 s) with the ICP elevation magnitude had a bi-exponential form whereas the same relationship of the total elevation time (2–20 s) was linear (Figure 4C). Neither ICP elevations nor relaxations were associated with significant changes to the heart or respiratory rates (Supplementary Table 1).

### Large-Volume Manual IT Administrations in Ported Monkeys

**FIGURE 5:**
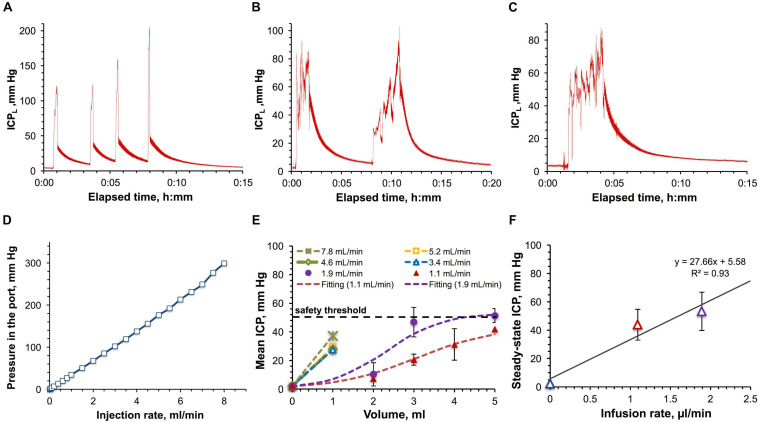
Large-volume bolus IT injections in monkeys. **(A)** Time-dependence of the ICP measured in-line (ICP_*L*_) for a series of 1 ml bolus injections in the cynomolgus monkey with the average infusion rate of 5.3 ± 1.8 ml/min. **(B)** Time-dependence of ICP_*L*_ for a series of 3 ml infusions in the cynomolgus monkey with the average infusion rate of 1.9 ± 0.6 ml/min. **(C)** Time-dependence of ICP_*L*_ for a 5 ml bolus injection in the rhesus monkey with the infusion rate of 1.8 ml/min. **(D)** Relation of the pressure in the IT injection port with the injection rate. **(E)** Dependence of the CSF pressure (ICP) on the infused volume for different infusion rates in the rhesus and cynomolgus monkey. The ICP was derived by correcting the ICP_*L*_ for the resistance pressure in the port. **(F)** Relation of the approximated steady-state ICPs with the infusion rates. Parameters of the linear regression are shown. Error bars on the panels **(E,F)** represent standard deviations.

From the translational perspective it was important to ascertain if the ICP effects observed in rats are similar to those in monkeys. In the pilot study, the impact of large-volume (with respect to the total CSF volume) IT infusions on the ICP was assessed by monitoring the ICP_*L*_ in the saline-filled catheter attached to the IT port during and after manual bolus administrations of 1, 3, and 5 ml (ca. 5–25% of the CSF volume) of saline in the cynomolgus (Figures 5A,B) and the rhesus monkey (Figure 5C). All injections caused the initial ICP_*L*_ elevation from the baseline level of 2.2 ± 0.7 mm Hg to 85–200 mm Hg. Once each injection was completed and a needle was withdrawn, ICP_*L*_ quickly decreased to 30–60 mm Hg followed by a subsequent significantly slower relaxation to the initial baseline ICP_*L*_ accompanied by pressure pulsations. Such a complex ICP_*L*_’s behavior can be explained by the fact that ICP_*L*_ is made up by two components–the endogenous CSF pressure and hydrodynamic resistance pressure in the injection system. The latter component can be further deteriorated by the partial catheter compression due to the tissue build-up around the catheter over time. However, the bottom estimate of the dependence of the resistance pressure on the infusion rate was obtained in the *in vitro* study using the same port and ICP_*L*_ measurement method (Figure 5D). The best linear fit was found for the 0–7 ml/min interval of injection rates. Time dependence of the ICP_*L*_ during infusion (Supplementary Figure 5A) at each tested rate from 0.01 to 8 ml/min demonstrates quick (2–13 s) establishment of the steady-state pressures characterizing hydrodynamic resistance. Relaxation to the baseline ICP after the infusion completion was somewhat slower and ranged from 8 to 21 s. Both elevation and relaxation durations were found to be a linear function of the infusion flow rate (Supplementary Figure 5B).

Characterization of the infusion resistance pressure in the port allowed assessment of the real pressure in the CSF. ICP dependence on the infused volume for the studied infusion rates was obtained by consolidating cynomolgus and rhesus monkey data and is shown in Figure 5E. Infusions with rates up to 2 ml/min revealed the tendency to lead to steady-state pressures, which were found to linearly depend on the infusion rates (Figure 5F), as in the rat studies. Over the entire duration of the procedures, the monkey’s vital parameters (heart and respiration rate, SpO_2_, CO_2_ content in the exhaled air, and rectal temperature) were constantly monitored. Respiration and cardiac complications (elevations of both rates followed by apnea) became apparent at pressures exceeding 50 mm Hg. Due to this safety limit, we were not able to formally characterize administrations of volumes higher than 1 mL for high-rate infusions (3.4–7.8 ml/min, Figures 5A,E).

As in the rat studies, the pressure relaxation kinetics in NHPs was found to be best fit by a bi-exponential curve (Supplementary Figure 6) as suggested by the bi-linear shape of the Guggenheim linearization (Supplementary Figure 7). Bi-exponentiality reflects the involvement of fast and slow relaxing components characterized by large and small time constants. Relative fractions of both components as a function of the ICP elevation magnitude are shown in Supplementary Figure 9B revealing the diminished contribution of the slow relaxation processes as the ICP increased. The total time of ICP relaxation from the peak value to the baseline level averaged 5.0 ± 0.6 min without a noticeable correlation with the ICP elevation magnitude.

## Discussion

This study was aimed at estimating the safety thresholds for IT infusions in rats and monkeys based on ICP-derived indices. In the first set of studies in rats, the animals were subjected to gradually increasing pressures applied within the CSF while measuring major ICP waveform characteristics, such as a mean ICP and a pulse amplitude of the fundamental component (P1, percussion wave). Analysis of the relationship between these two characteristics is frequently used for the assessment of the ICP autoregulatory capacity and the compensatory reserve (Balestreri et al., 2004; Czosnyka and Pickard, 2004; Kawoos et al., 2015). These concepts characterize the physiological mechanisms of adaptation to the increased ICP. Exhaust of these resources is typically associated with irreversible changes due to the high pressures and leads to poor outcomes (Czosnyka and Pickard, 2004; Kawoos et al., 2015).

### ICP Safety Thresholds

Published analytical (Marmarou et al., 1975) and experimental (Avezaat et al., 1979) data demonstrate a linear relationship between the ICP pulse amplitude (P1) and the mean ICP. Additionally, detailed analysis of the sigmoid form of the ICP-CSF volume curve suggests three different degrees of linearity specific for three different ICP zones (Avezaat et al., 1979; Balestreri et al., 2004; Czosnyka and Pickard, 2004). The first zone, situated at low pressures, is characterized by low synchronization between changes in amplitude and a mean ICP, indicating a good compensatory reserve used to accommodate volume alterations occurring as a result of cerebral blood pulsations. In the middle zone, this synchronization is more pronounced and therefore the pulse amplitude increases more readily with the raised ICP. This denotes a poor compensatory reserve, implying that even small volume augmentations can become uncompensated trending toward rapid ICP elevations that can become dangerous in nature. Negative correlation at high ICPs denotes detrimental, irreversible changes in the compensatory mechanisms leading to permanent structural damages or death.

#### Rats

Our rat data (Figure 1D) is consistent with the described amplitude-pressure paradigm showing a relatively broad (18 mm Hg) area of low synchronization and similar middle and third zones (ca. 10 mm Hg-wide each). By combining the first and second zones, one can derive the working range of safe ICPs for performing IT administrations. This relatively wide range spans from the baseline level of 8.6 ± 1.7 mm Hg through the critical ICP of 27.5 ± 3.8 mm Hg. Animals that underwent pressures higher than the critical ICP for prolonged periods of time (10–15 min) experienced a fatal outcome likely associated with cardiac complications. In fact, during ICP elevation we observed a progressive shift of the fundamental (P1) component of the ICP pulse wave to higher frequencies (from 5.4 to 8.3 Hz) (Figure 1C) indicating an increased heart rate (Supplementary Figure 8).

#### NHPs

Inability to observe ICP waveform and therefore measure mean ICP amplitude through the injection port in NHP was the major limitation for employing the same approach for defining the ICP safety threshold as in rats. Therefore, we relied only on the physiological response to elevated ICPs. Very consistently across all the tests, reaching the ICP of 50 mm Hg rapidly caused respiratory and cardiac complications (elevations of both rates followed by apnea) demanding resuscitation interventions. Given reproducible stability of vital physiological measurements at ICPs below 50 mm Hg, we assumed this level as a tentative safety threshold to be further validated using orthogonal methods and in a larger group of animals.

The safety thresholds in rats and NHPs were found to be in a good agreement with low- and fast-rate infusion tests when put in the context of our prior research and the data of others as further discussed.

### Low-Rate Infusions: The Role of CSF Drainage

As a criterion of tolerance for elevated ICPs, it was proposed (Jones et al., 1987) to use a linearity of the plateau ICP-infusion rate relationship derived from a constant-rate infusion experiment. It was suggested (Jones et al., 1987) that deviations from linearity at high rates, in the form of lower plateau ICP than expected (decrease in the CSF outflow resistance) could be indicative of altered flow characteristics of the CSF outflow sites. Most of these sites are located on the CSF-blood interface in the arachnoid protrusions (arachnoid villi) within the venous sinuses in the cranial (superior sagittal and transverse venous sinuses) and spinal (venous sinuses near dorsal root ganglia) compartments (Kapoor et al., 2008; Pollay, 2010). At high flow rates, unidirectional and CSF pressure-dependent (Pollay, 2010) flow through arachnoid villi can reach intrinsic capacity thereby hypothetically increasing the risk of structural damages to the drainage components. For instance, ultrastructural changes in the arachnoid villi of adult rats have been reported for CSF pressures above 22 mm Hg (Butler et al., 1983). A linear plateau pressure response to constant-rate infusions with maximal ICP ranges from 15 to 30 mm Hg, has been found for a number of adult animals [mice (Jones, 1985), rats (Jones et al., 1987), cats (Sullivan et al., 1979), rabbits (Davson et al., 1970), monkeys (Blasberg et al., 1981)] and man (Cutler et al., 1968; Ekstedt, 1977; Albeck et al., 1991). In one study, a non-linear response has been observed in rats even at low infusion rates (<34 μl/min) although the plateau ICP ranged from 29 to 81 mm Hg (Jones et al., 1987). At rates higher than 34 μl/min, equilibrium pressures were not consistently obtained, and electroencephalographic patterns began to reflect the disturbed cerebral function. Therefore, acquisition of steady-state pressures might be considered a criterion of physiologically safe adaptation to the slowly infused liquids regardless of the shape of ICP-infusion rate relationship. This is consistent with our findings in rats and NHPs exhibiting plateau ICPs only below safety thresholds (Figures 3A, 5E).

Considering that a constant-rate infusion method is associated with forced perfusions through the leptomeningeal space, which could hypothetically be damaging *per se* at high rates, we developed the alternative method, which harnessed a direct measurement of the rate of unconstrained, free drainage at varying ICP values (Figure 2). The obtained gross exponential form of the drainage rate-ICP relationship suggests a high throughput of the CSF drainage system. We hypothesize the involvement of several contributary factors activating at different pressures, which is consistent with recent findings of other authors (Vinje et al., 2020). A good linear regression obtained until the ICP of 23 ± 1 mm Hg can be attributed to drainage through the arachnoid villi based on the previously reported data (Jones et al., 1987) and correlation with the linear region in the constant-rate infusion study (Figure 3B). However, further deviations from linearity occurring from 23 ± 1 mm Hg to 30 ± 3 mm Hg (MTP) in Figure 2B and similarly from 23.5 to 28.8 mm Hg (critical pressure) in Figure 3B are likely explained by the emergence of additional, extra-villous drainage pathways rather than previously suggested pressure-related ultrastructural damages to arachnoid villi (Butler et al., 1983). Indeed, deviations from linearity were still characterized by the steady state ICP condition at the respective pressures (Figure 3A), which is suggestive of physiologically normal functionality of the underlying mechanisms. Furthermore, the ICP relaxation process repeatedly returned the elevated ICP to its baseline value (Figure 3C), albeit at faster rates (Figure 3D), which would not have been the case if any damages had occurred. Additionally, it should be noted that the entire interval of tolerated pressures (6–30 mm Hg) in Figure 2B correlates with the range of unimpaired compensatory mechanisms (9–28 mm Hg) in the constant pressure study (Figure 1D). Mortality at pressures averaging 30 ± 3 mm Hg occurred only after prolonged (>10 min) exposures and is likely explained by extended, non-elastic (as suggested by negative correlation in the respective ICP region on Figure 1D) compressions of vital CNS or vascular components (requires further investigation). The following reduction of the drainage rate at ICP > MTP is likely indicative of the discontinuation of some drainage pathways due to death.

The exact mechanisms of the CSF drainage through the mesothelial layer of arachnoid villi are insufficiently elucidated due to significant methodological difficulties. The working hypotheses include an active vesicular transport (Butler et al., 1977) such as a fluid-phase caveolae-mediated macropinocytosis (Tuma and Hubbard, 2003; Falcone et al., 2006) and filtration through either permanent or dynamic mesothelial pores formed by merged vacuoles (Tripathi and Tripathi, 1974). A recent study of a new fluid drainage mechanism resembling a pressure relief valve in the endolymphatic sac of the inner ear (Swinburne et al., 2018) highlights a lot of similarities in the regulation of fluid traffic in various tissues (Tripathi and Tripathi, 1974; Gong et al., 2002; Møller et al., 2013). This adds the theoretical possibility of a similar passively working drainage pathways in the mesothelial layer of arachnoid villi. Figure 2B might be suggestive of the collaborative work of active and passive drainage mechanisms. In fact, average post-mortem reduction of the unconstrained drainage can be explained by the dysfunction of active processes and the remaining functionality of the drainage components that are based solely on physical principles (permanent pores). Research into the relevance and relative contributions of the proposed mechanisms remains in high demand.

Extra-villous CSF absorption pathways might also contribute to the fluid accommodation and pressure maintenance. However, their role and the location remain poorly investigated. The most widely discussed route is the hypothesized connection between the CSF and the lymphatic system (Koh et al., 2005). The amount of the CSF drained through this pathway is highly debatable, varying from negligible or little (Marmarou et al., 1994; Gillooly et al., 2017) to quite significant (up to 50%) (Boulton et al., 1999; Ma et al., 2017). Our studies utilizing PET imaging unequivocally demonstrated species-dependence of this process, with larger fraction of the CSF drained in rats 2.4 ± 1.8%) than in NHP (0.32 ± 0.28%) (Gillooly et al., 2017; Papisov and Belov, 2019). Whatever the fraction of CSF is drained at normal CSF pressure, elevated ICP might potentially increase it (Boulton et al., 1998; Silver et al., 1999). Given a lack of human data, attention should be paid when extrapolating the pressure data between species.

Overall, the data obtained in this study for low-rate infusions is consistent with our previous imaging studies in rats (Belova et al., 2012; Papisov et al., 2012b; Gillooly et al., 2017) and monkeys (Calias et al., 2011; Papisov et al., 2012a, 2013; Gillooly et al., 2017; Papisov and Belov, 2019). Specifically, quantification of the clearance pathways of various macromolecular substances injected IT as a slow bolus suggested the primary role of CSF drainage mechanisms in adaptation to added volumes and raised ICP. The resulting equilibration of the infusion rate and the pressure-dependent drainage rate prevents liquid and pressure buildup and enables continuous perfusion within the leptomeningeal space. We found that in rats all infusions leading to the plateau pressures could safely be used for IT administration of at least 240 μl [ca. 100% of the CSF volume in rats (Jones et al., 1987)]. Although the upper volume limit remains to be determined, it is likely to depend more on the compound’s cytotoxicity and hemoconcentration rather than volumetric limits of the leptomeningeal space. This observation lays the groundwork for the development of the CSF dialysis technology, which has the potential to become a novel treatment method as noted recently (Johanson et al., 2008).

### High-Rate Infusions: The Role of Craniospinal Compliance

High-rate infusions and bolus injections (2.7 ± 0.9 ml/min in rats and 5.3 ± 1.8 ml/min in monkeys) caused rapid uncompensated fluid buildup and pressure elevations, which triggered different physiological reactions in rats and NHPs. The upper ICP threshold for bolus injections in rats was 200 mm Hg, which corresponded to a bolus volume of 1.5 ml (Figure 4B). Short exposure time appeared to play a critical role in the tolerance of these pressures in rats, as no respiratory or heart rate changes were observed (Supplementary Table 1). In monkeys, the upper ICP threshold was limited to 50 mm Hg regardless of the exposure time and was related to immediate respiratory and cardiac complications (elevations of both rates followed by apnea) demanding resuscitation interventions. The cardiac complications might be somewhat attributed to the partial compression of cerebral vessels. Our recent study (Pass et al., 2018) showed that in monkeys, the vascular factor contributed ca. 10% to compliance at pressures higher than the diastolic pressure (30–50 mm Hg under isoflurane anesthesia). More studies in a larger group of animals will be needed to refine the measured parameters as well as to more accurately establish the underlying mechanisms.

Acute uncompensated ICP elevations suggest the substantially diminished contribution of the drainage system and the primary role of the components of craniospinal compliance in adaptation to high-rate bolus administrations. These components involve mechanical entities capable of limited expansion and contraction. Such physical factors of adaption contribute to the fast ICP elevation (<20 s) and relaxation (<200 s) times (Figure 4C) suggesting low elasticity capacity of the involved components. This contrasts with physiological factors (drainage systems), for which significant activation times (1–15 min) were observed in rats (Figure 3A). Although the exact locations of the entities contributing to the hydrostatic compliance have not yet been established, these hypothetically include membranes of the atlanto-occipital and atlanto-axial joint capsules, membranes covering openings (foramina), through which nerves and blood vessels exit the leptomeningeal space, cerebral and spinal nerve sheaths, and fluid compartments of the inner ear. Whereas enlargement of the optic nerve’s meningeal sheath at elevated ICP is well documented (Hansen and Helmke, 1997; Watanabe et al., 2008), the behavior of other nerves’ sheaths is yet to be established. In rodents, the nerve bundles exiting the cribriform plate are significantly more developed than those in primates and therefore their contributions to volume and pressure attenuations may be expected at a higher degree. The role of the inner ear might be mediated by the established communication via the cochlear aqueduct between the subarachnoid space of the posterior cranial fossa and the perilymphatic space of the cochlea. Widely open in most mammals and variably patent in humans [89% of young adults and 70% of older adults (Salt and Hirose, 2018)], the cochlear aqueduct is situated in the petrous part of the temporal bone and therefore is incapable of any expansions. This rigid structure facilitates instant pressure transmission from the CSF to the fluids of the inner ear (Thalen et al., 2001). However, compliant cochlear membranes (oval and round windows, tympanic, Reissner’s and basilar membranes) can plausibly accommodate some minor volume and pressure changes. Such compliance is believed to be critical for reducing the risk of structural damages to the cochlea due to relatively large and rapid ICP changes as a result of everyday events such as coughing and sneezing (Marchbanks and Reid, 1990). These functions of intra-cochlear hydromechanics underlie the novel emerging method of non-invasive ICP monitoring based on the measurements of the tympanic membrane displacement (Wunderlich et al., 1996).

### ICP Relaxation

How a system returns to the initial state after perturbations is an important parameter of its adaptive capacity and can also be indicative of the involved mechanisms. In this respect, ICP relaxation to the baseline level after low- and high-rate infusions was analyzed in rats and monkeys. Low-rate infusions characterized by the steady-state ICP demonstrated the longest pressure relaxation durations (141–1047 s in rats and 300 ± 36 s in monkeys). This observation is consistent with the primary contribution of the slow-responsive CSF drainage system to the adaptation to this mode of administration. However, this contribution was gradually diminished with the increase of the infusion rate as indicated by the distortion of the shape of the relaxation curve by the appearance of the quick-relaxation phase (Phase I on Figure 3C). Analysis of the purely bi-exponential relaxation curves specific for bolus injections in rats (Supplementary Figures 3, 4) and monkeys (Supplementary Figures 6, 7) clearly revealed the ever increasing role of the rapidly relaxing component with the raise in the ICP elevation magnitude (Supplementary Figure 9). This component can likely be attributed to the mechanisms of craniospinal compliance, very limited elasticity capacity of which explains the fast ICP elevation (2–20 s) and relaxation (9–172 s) times after bolus injections in rats. The gradual transition from the leading role of CSF drainage to compliance mechanisms was also illustrated by the change of the respective relationships between the relaxation time and the ICP elevation magnitude. Whereas it is linear at low-rate infusions (Figure 3D), it is reciprocally bi-exponential at high rates (Figure 4C), with the transition occurring within the 15–20 mm Hg region.

### Interspecies Differences

Comparison of the volume and pressure tolerance characteristics in rats and monkeys reveals many similarities, though, it is essential the differences be emphasized. The same shape of the pressure-volume curves at low infusion rates suggests common underlying CSF drainage mechanisms. However, in rats these mechanisms could possibly be supplemented with lymphatic (or other) drainage pathways. Increased cardiac and respiratory rates at ICPs approaching, or exceeding, the safety thresholds suggest the involvement of vascular reactivity, which in rats was significantly slower as evidenced by the time dependence of adversity. However, the most dramatic differences were observed in the tolerance of pressures exceeding the safety thresholds, which was specific for large-volume bolus injections. These pressures in monkeys (>50 mm Hg) immediately caused apnea, likely explained by the excessive pressure on the respiratory center in the medulla oblongata and pons. This effect in rats was not observed until the ICP of 200 mm Hg, which suggests fundamental physiological differences critical for considering inter-species data extrapolation. Given the phylogenetic relationship between NHPs and humans, as well as similar baseline and critical pressures and CSF drainage mechanisms, one can expect similar ICP tolerance patterns. However, infusion rate patterns are likely to differ to the extent determined by variations in total CSF volume, CSF clearance sites abundance, and drainage performance to be explored in further clinical tests.

### Limitations

The major limitation of the current study is a small size of the animal groups and the pilot character of the NHP tests. Therefore, the results reported here are anticipated to guide further research into refining the numerical values of established thresholds as well as underlying mechanisms.

Although the CSF access during all the injections was controlled using various methods, CSF outflow pathways were not directly measured or visualized. This limitation was partially addressed by discussing the obtained results in the context of our prior PET/CT imaging studies in rats (Belova et al., 2012; Papisov et al., 2012b; Gillooly et al., 2017) and NHPs (Calias et al., 2011; Papisov et al., 2012a, 2013; Gillooly et al., 2017; Papisov and Belov, 2019). In those studies, the clearance of various radiolabeled substances from the CSF was quantitatively characterized at various time points after the bolus IT administrations similar to those reported here. The concurrent utilization of ICP monitoring and CSF imaging methods would be beneficial in further research.

Inability to observe ICP waveform and therefore measure mean ICP amplitude through the injection port in NHP was the major obstacle for employing the same approach for defining the ICP safety threshold as in rats, i.e., determined by the transition from the zone of poor compensatory reserve to the zone of impaired compensation on the ICP amplitude–mean ICP relationship. More invasive studies enabling positioning of the pressure transducer in the CSF pool would be beneficial for refining the obtained results.

## Conclusion

Intracranial pressure monitoring is a valid method for determining safety thresholds for IT therapy delivery. Various mechanisms, such as craniospinal compliance and CSF drainage to the blood, play role in adaptation to elevated CSF pressures. However, their relative contributions, capacity, and physiological correlates are species-dependent and therefore caution should be used in extrapolation of the results between species. As such, successful clinical translation of large-volume IT methodologies will require follow-up tests in humans. We anticipate our data to be instrumental for planning and conducting such tests as well as pre-clinical trials of IT drug candidates. It is clear that volume and infusion rates define the duration and magnitude of CSF pressure elevation and both factors must be considered in the optimization of delivery strategies with respect to safety thresholds. While very limited elasticity capacity of the components of craniospinal compliance facilitates solute distribution in the CSF, it is important to pay attention to not exceed the safety thresholds at high-rate administrations. Notably, a pathological process or trauma may impact any of those thresholds and therefore the disease’s potential influence should be considered each time IT therapy delivery is to be used. Availability of methodologies enabling fast and reliable assessment of safety thresholds would significantly facilitate both pre-clinical research and, in a clinical setting, development of delivery strategies optimized for individual patients from both efficacy and safety perspectives.

## Data Availability Statement

The raw data supporting the conclusions of this article will be made available by the authors, without undue reservation.

## Ethics Statement

The animal studies were reviewed and approved by Institutional Animal Care and Use Committee (IACUC) of Massachusetts General Hospital.

## Author Contributions

VB conceived, designed, and performed all the rat studies, performed ICP measurements in NHPs, analyzed and interpreted all ICP data, and wrote the first draft of the manuscript. JA assisted with the development and carrying out of ICP measurements in rats, performed NHP studies, and analyzed the ICP data. SL performed ICP measurements in rats and analyzed the data. PG and BD assisted with ICP procedures and data analysis. MP conceived and designed NHP studies, assisted with procedures, and interpreted the data. All authors contributed to manuscript revisions.

## Conflict of Interest

The authors declare that the research was conducted in the absence of any commercial or financial relationships that could be construed as a potential conflict of interest.
