# Supplementary material for: Large-Volume Intrathecal Administrations: Impact on CSF Pressure and Safety Implications
Source: Front Neurosci. 2021 Apr 14;15:604197. doi: 10.3389/fnins.2021.604197 (PMC8079755; doi:10.3389/fnins.2021.604197)
Supplement: Supplementary file 1 [file Data_Sheet_1.PDF]

Supplementary Material

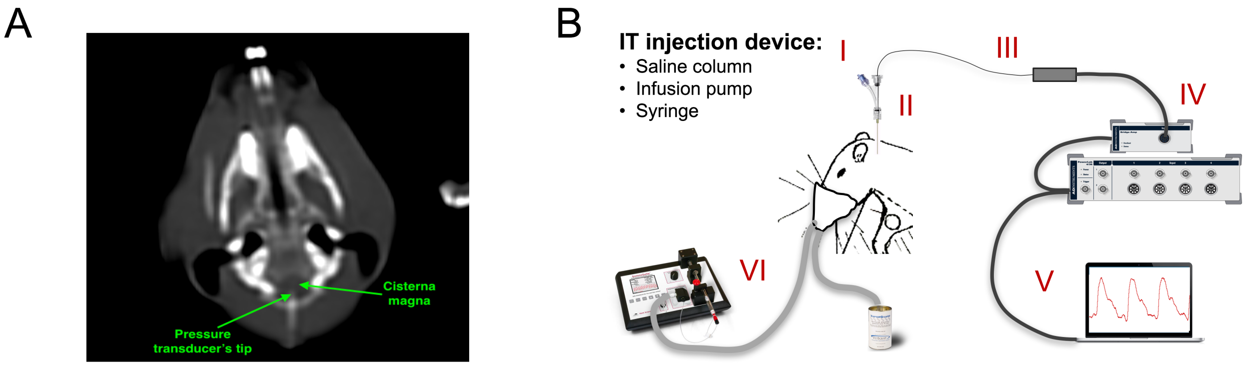

Supplementary Figure S1. ICP measurements in rats. **(A)** Post-mortem CT scan of the atlanto-occipital junction verifying the catheter positioning in the cisterna magna. **(B)** A sketch of the equipment configuration, which includes the following components: (I) IT injection device (saline bag set at different heights, infusion pump, or syringe for manual injections), (II) Tuohy Borst adapter attached to the catheter inserted into the cisterna magna, with injection device connected to the adapter's side arm, (III) micro-tip pressure sensor advanced into the cisterna magna through the adapter and catheter, (IV) data acquisition platform, (V) control and data analysis console, (VI) small-animal isoflurane anesthesia platform.

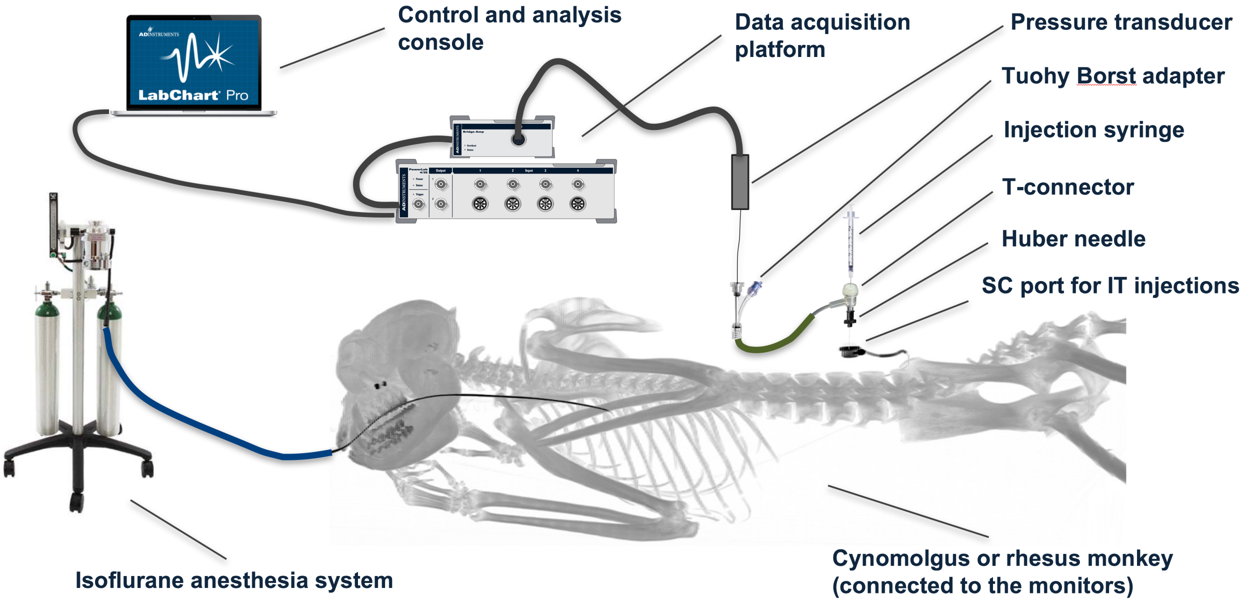

Supplementary Figure S2. ICP measurements in monkeys. A sketch of the equipment configuration.

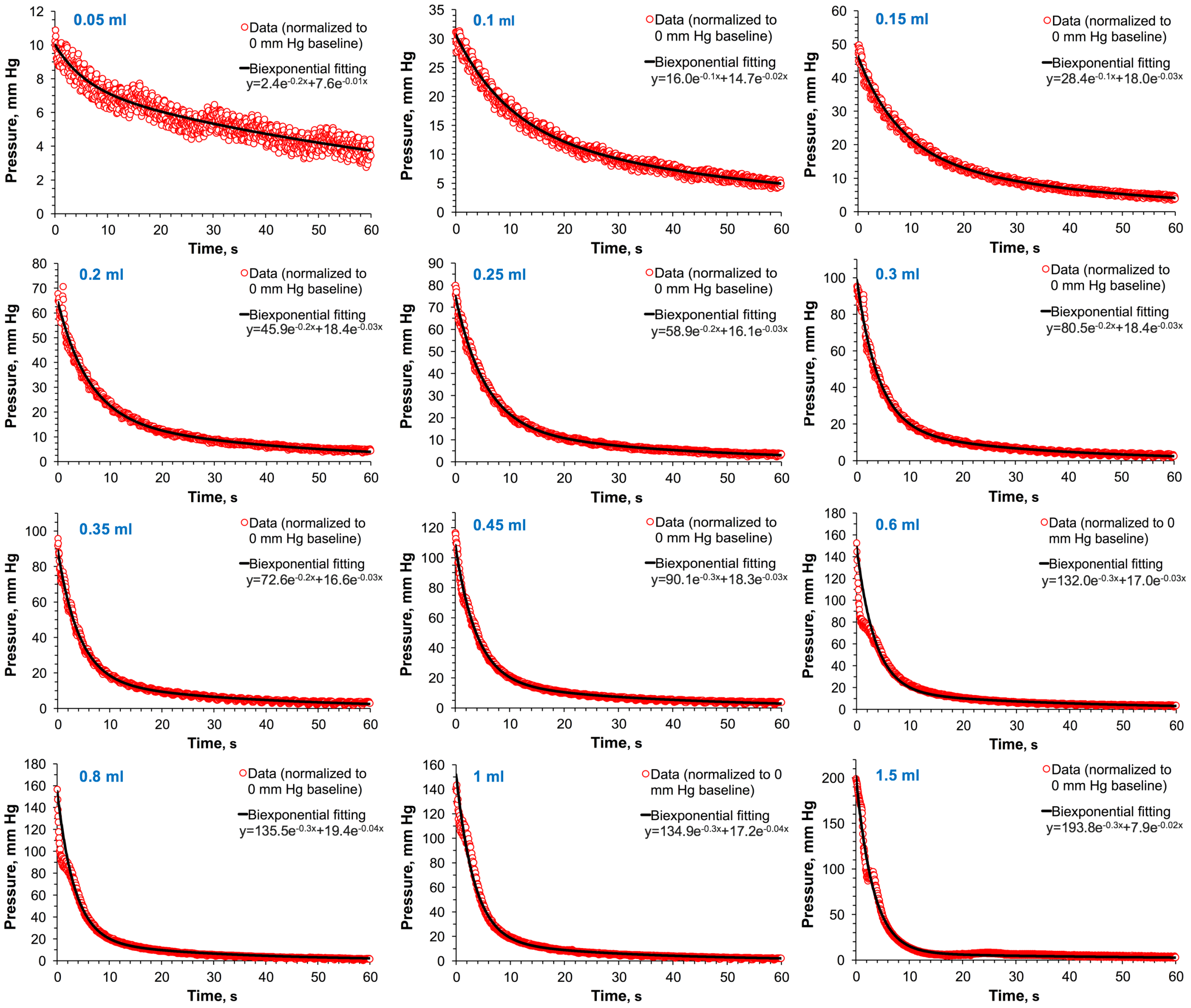

Supplementary Figure S3. ICP relaxation to the baseline value after bolus IT injections in the cisterna magna of a rat. Left to right: time-dependence curves of ICP (blue dots) and their bi-exponential fitting (red lines) after reaching the peak ICP as a result of administrations at 2.7±0.9 ml/min of 0.05 to 1.5 ml of saline. A time interval of 60s is shown. All data are normalized to 0 mm Hg baseline to account for differences in resting pressures.

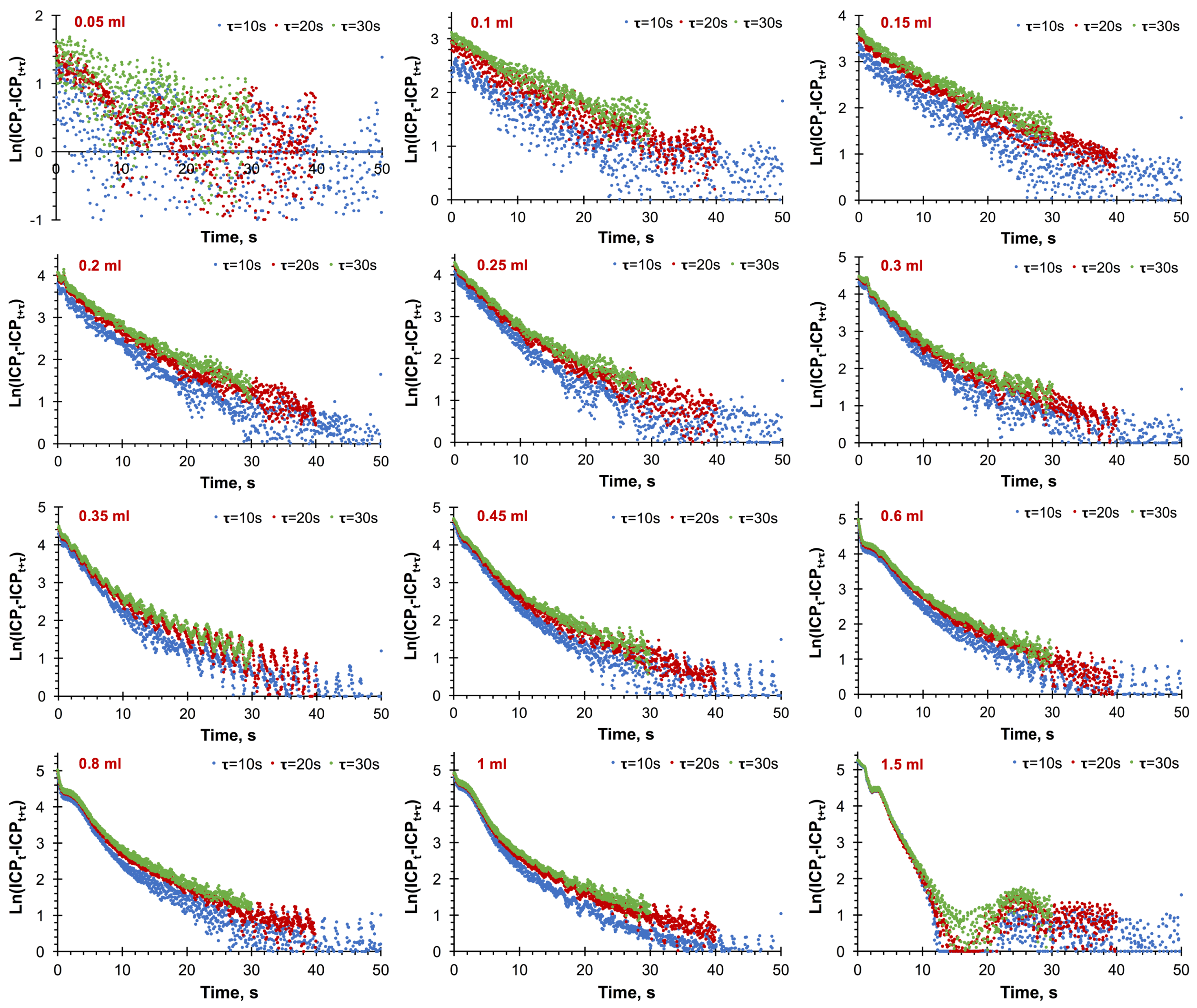

Supplementary Figure S4. Guggenheim linearization of the ICP relaxation after bolus IT injections in rats. Left to right: time dependence of the logarithm of difference between two ICP values on the ICP relaxation curves (**Supplementary Figure S1**), which are 10 s (blue dots), 20 s (red dots), and 30 s (orange dots) apart, after administrations at 2.7±0.9 ml/min of 0.05 to 1.5 ml of saline. A time interval of 50 s is shown. Bi-linear shape is indicative of bi-exponential character of the ICP relaxation.

	Elevation		Relaxation	
Volume, ml	Heart rate, bpm	Respiration rate, bpm	Heart rate, bpm	Respiration rate, bpm
0.05	293	60	293	60
0.10	234	60	234	60
0.15	234	60	234	60
0.20	234	60	234	60
0.25	234	60	234	60
0.30	234	60	294	60
0.35	294	60	354	60
0.45	354	60	354	60
0.60	354	60	354	60
0.80	354	60	354	60
1,00	354	60	354	60
1.50	354	60	294	60

Supplementary Table S1. The effect of bolus IT injections on the heart and respiration rates in rats. Heart and respiration rates in beats per minute (bpm) are provided for different injected volumes (0.05-1.5 ml) during ICP elevation and relaxation phases.

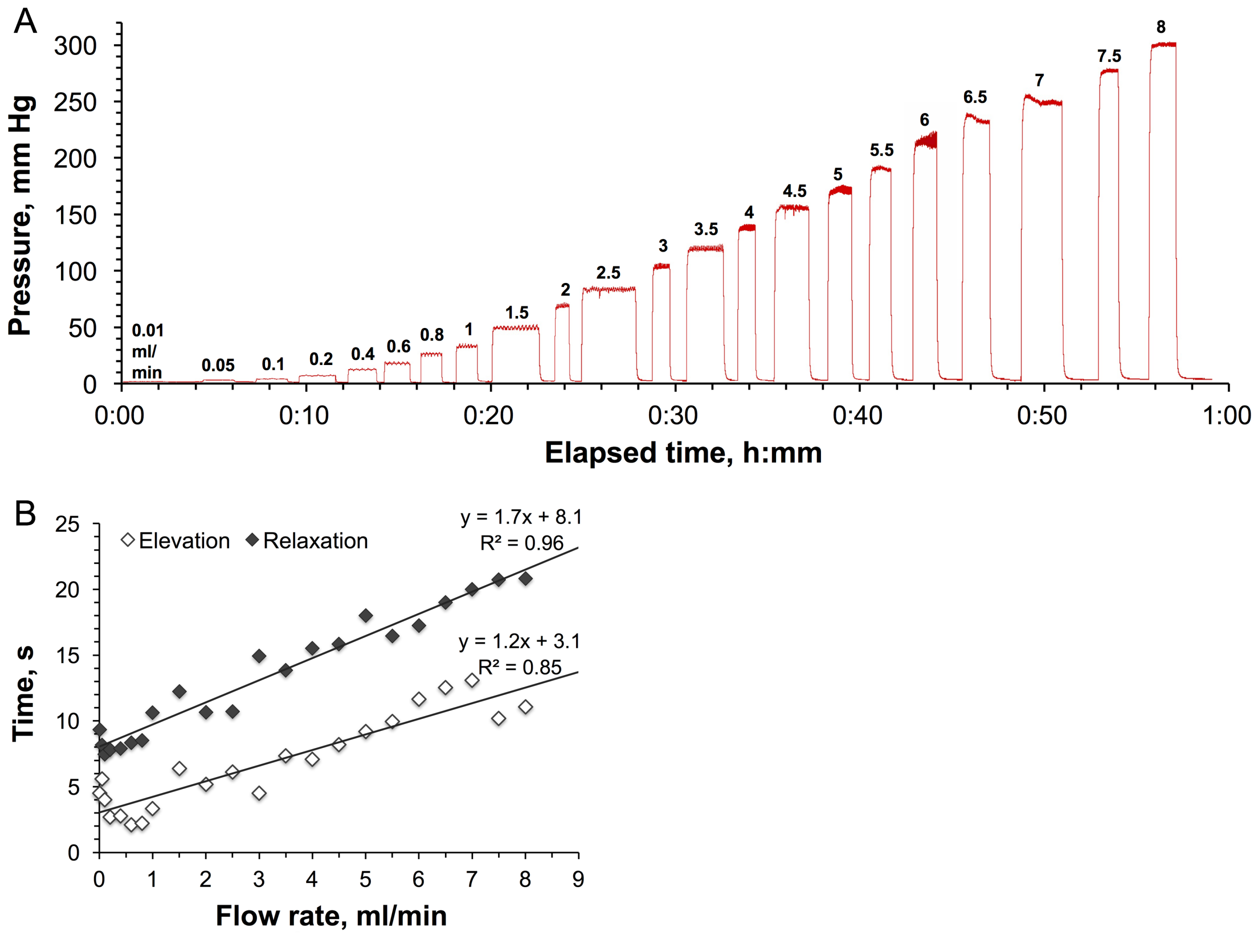

Supplementary Figure S5. Characterization of the pressure resistance in the IT injection port in monkeys. **(A)** Time dependence of pressure in the IT port measured ex-vivo for various injection flow rates from 0.01 ml/min to 8 ml/min. **(B)** Duration of the pressure elevation and relaxation phases as a function of the injection flow rate.

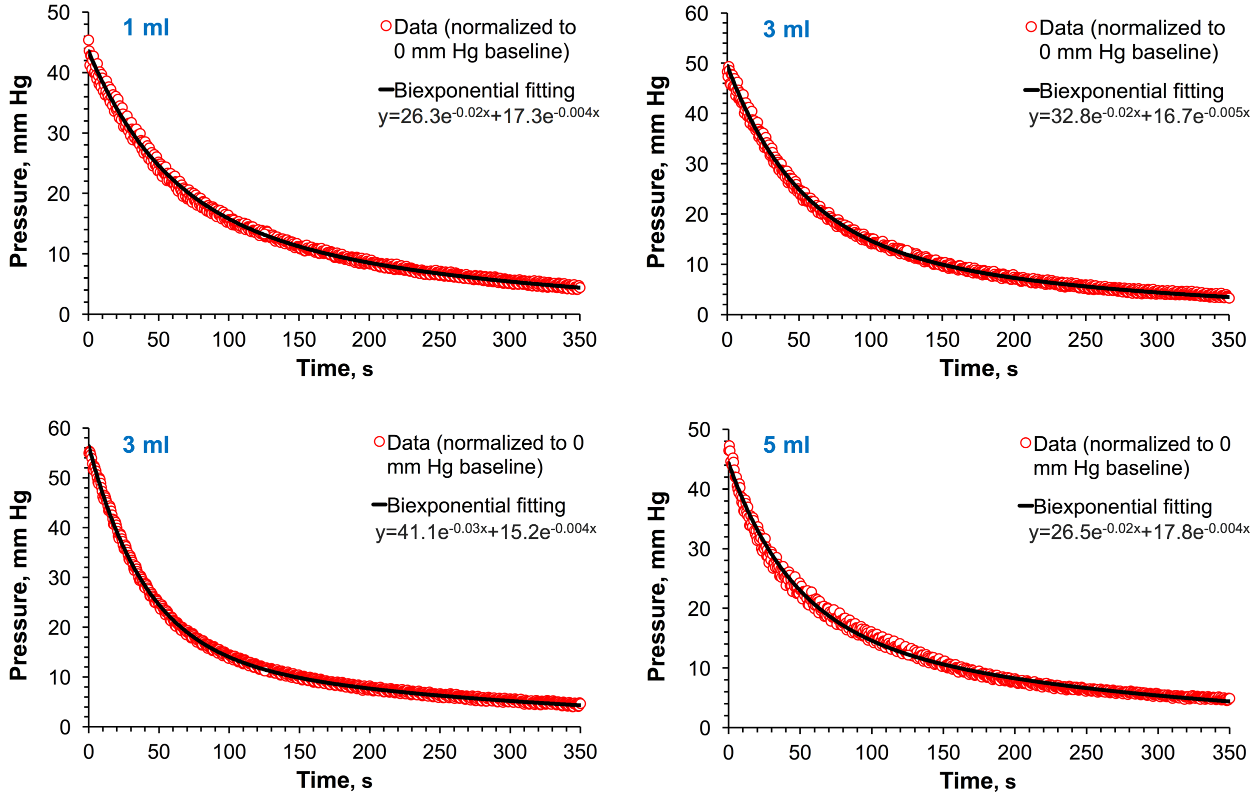

Supplementary Figure S6. ICP relaxation to the baseline value after bolus IT injections in the lumbar ports of monkeys. Left to right: time-dependence curves of ICP (blue dots) and their bi-exponential fitting (red lines) after reaching the peak ICP as a result of administrations at 4.8±1.5, 1.7±0.5, and 2.1 ml/min of 1, 3, and 5 ml of solutes, respectively. All the data are normalized to 0 mm Hg baseline to account for differences in resting pressures.

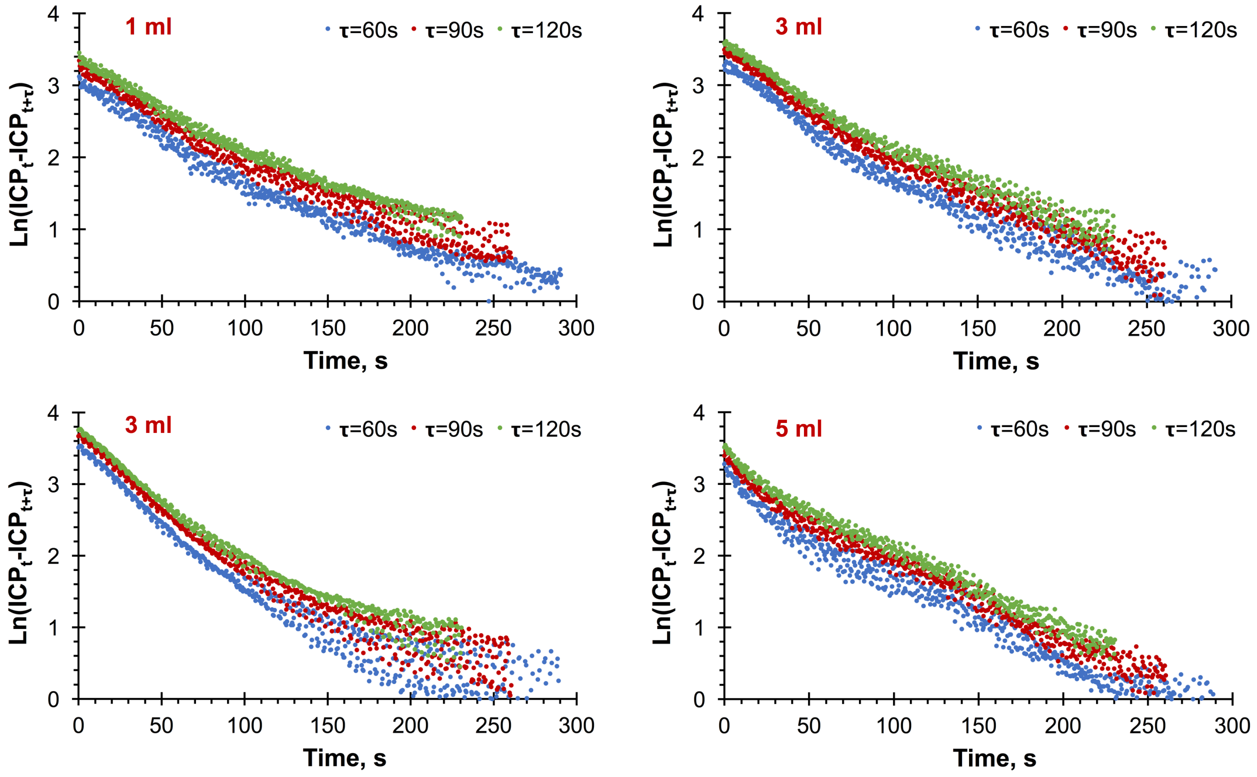

Supplementary Figure S7. Guggenheim linearization of the ICP relaxation after bolus IT injections in the lumbar ports of monkeys. Left to right: time dependence of the logarithm of difference between two ICP values on the ICP relaxation curves (Fig. S4), which are 60 s (blue dots), 90 s (red dots), and 120 s (orange dots) apart, after administrations at 4.8±1.5, 1.7±0.5, and 2.1 ml/min of 1, 3, and 5 ml of solutes, respectively. A time interval of 300 s is shown. Bi-linear shape is indicative of bi-exponential character of the ICP relaxation.

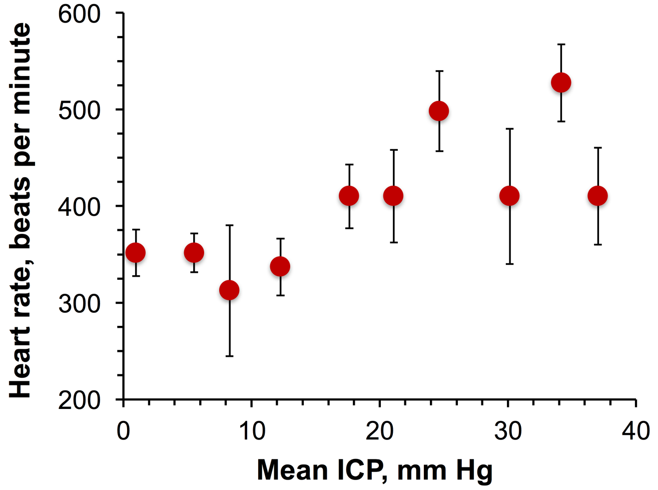

Supplementary Figure S8. Dependence of the heart rate on the ICP caused in rats for extended durations (>10 min). The ICP was measured directly in the cisterna magna. The heart rate was obtained by conversion of the P1 frequency component of the ICP pulse wave into beats per minute.

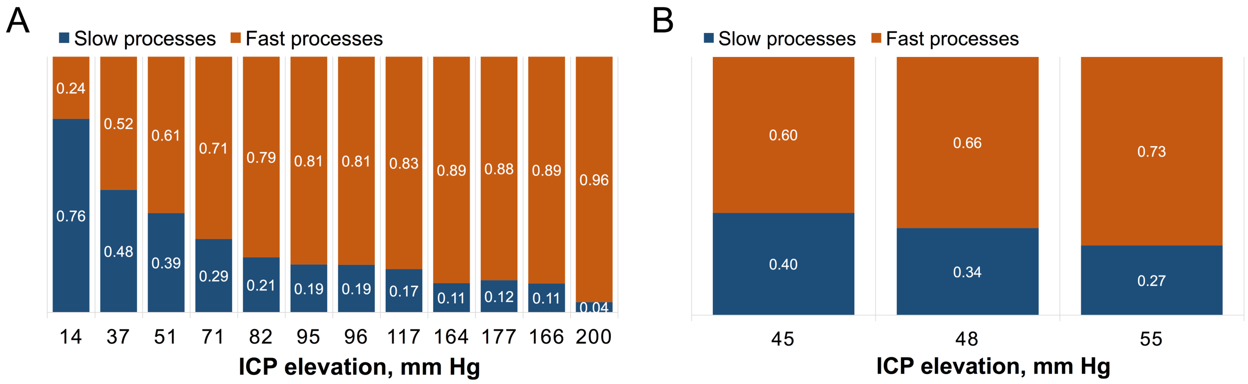

Supplementary Figure S9. Contribution of the slow and fast processes to the ICP relaxation. The diagrams consist of the relative fractions of the slow (navy) and fast (orange) components of the bi-exponential function used to fit the ICP relaxation curves (**Supplementary Figures S3** and **S6**) after bolus injections in rats **(A)** and monkeys **(B)**. The fractions were obtained based on the pre-exponential coefficients. The slow and fast components of a bi-exponential function are characterized by longer and shorter half-lives, respectively.
